# Alcohol is not associated with increased mortality in adolescent traumatic brain injury patients

**DOI:** 10.1007/s00383-021-05057-z

**Published:** 2021-12-27

**Authors:** Zachary N. Lu, Eric O. Yeates, Areg Grigorian, Russell G. Algeo, Catherine M. Kuza, Theresa L. Chin, Megan Donnelly, Allen Kong, Jeffry Nahmias

**Affiliations:** 1 Division of Trauma, Burns and Surgical Critical Care, Department of Surgery, University of California, Irvine, 333 City Blvd. West, Suite 1600, Orange, CA 92868-3298 USA; 2 Department of Surgery, University of Southern California (USC), 1520 San Pablo St., Suite 4300, Los Angeles, CA 90033 USA; 3 Department of Anesthesiology, Keck School of Medicine, University of Southern California, 1250 San Pablo St., Suite 3600, Los Angeles, CA 90033 USA

**Keywords:** Adolescent, Traumatic brain injury, Alcohol, Mortality, Outcomes, Database

## Abstract

**Purpose:**

Compared to adults, there is a paucity of data regarding the association of a positive alcohol screen (PAS) and outcomes in adolescent patients with traumatic brain injury (TBI). We hypothesize adolescent TBI patients with a PAS on admission to have increased mortality compared to patients with a negative alcohol screen.

**Methods:**

The 2017 Trauma Quality Improvement Program database was queried for patients aged 13–17 years presenting with a TBI and serum alcohol screen. Patients with missing information regarding midline shift on imaging and Glasgow Coma Scale (GCS) score were excluded. A multivariable logistic regression analysis for mortality was performed.

**Results:**

From 2553 adolescent TBI patients with an alcohol screen, 220 (8.6%) had a PAS. Median injury severity scores and rates of penetrating trauma (all *p* > 0.05) were similar between alcohol positive and negative patients. Patients with a PAS had a similar mortality rate (13.2% vs. 12.1%, *p* = 0.64) compared to patients with a negative screen. Multivariate logistic regression controlling for risk factors associated with mortality revealed a PAS to confer a similar risk of mortality compared to alcohol negative patients (*p* = 0.40).

**Conclusion:**

Adolescent TBI patients with a PAS had similar associated risk of mortality compared to patients with a negative alcohol screen.

## Introduction

Traumatic brain injuries (TBI) are common among adolescents and result in over 1 billion dollars in hospital-related costs annually, as well as substantial morbidity and mortality [1, 2]. Alcohol use is identified in up to 5% of traumatically injured adolescent patients [3]. Understanding the impact of alcohol in adolescents suffering from TBI may be useful in predicting outcomes in this population.

Alcohol’s impact on mortality and other outcomes in adult TBI has been extensively studied. Existing studies have had mixed results, with some reporting lower mortality in alcohol positive patients [4–8], while others demonstrating similar or higher mortality compared to alcohol negative patients [6, 9–15]. Reported results on the effects of other outcomes including hospital length of stay (LOS), intensive care unit (ICU) LOS, and ventilator days between alcohol positive and negative patients are also variable [4, 5, 7, 9, 11, 12, 14, 16, 17]. A single national database study in adolescent trauma patients showed higher risk of mortality and hospital complications including pneumonia and sepsis for patients with a positive alcohol screen, but did not specifically focus on patients with traumatic brain injury [18].

This study aimed to investigate the effects of alcohol on adolescent patients sustaining TBI, hypothesizing that adolescent TBI patients with positive alcohol screens on admission would have increased mortality, complications, and LOS compared to adolescent TBI patients with negative alcohol screens.

## Materials and methods

The 2017 Trauma Quality Improvement Program (TQIP) database was queried for all patients between the ages of 13 and 17 years old who sustained a TBI (utilizing ICD-10 codes 800–801.96, 803–804.99, 850–854.19) and had a documented alcohol screen on admission. The TQIP database includes data collected from over 700 trauma centers from across the United States. Data quality and accuracy is frequently assessed and optimized via validation programs that measure errors and identify portions of the database that require further assessment [19]. Patients with missing information regarding the presence/absence of midline shift on head computed tomography (CT) imaging or Glasgow Coma Scale (GCS) score on admission were excluded. Midline shift was defined in TQIP as > 5 mm shift of the brain past its center line within 24-h after time of injury. Patients were placed into two groups, patients with a positive alcohol screen and patients with a negative alcohol screen. The use of this database was approved by the Institutional Review Board of the University of California, Irvine and deemed exempt from need for written informed consent as it utilized a deidentified national database. This manuscript was prepared using the pertinent Strengthening the Reporting of Observational Epidemiology (STROBE) Statement guidelines.

The primary outcome was mortality. The secondary outcomes included hospital LOS, ICU LOS, ventilator days, and complications including unplanned ICU admission, unplanned intubation, unplanned return to the operating room, pneumonia or ventilator-associated pneumonia (VAP), organ space surgical site infection (SSI), superficial SSI, catheter-associated urinary tract infection (CAUTI), central line-associated bloodstream infection (CLABSI), sepsis, deep vein thrombosis (DVT), pulmonary embolism, and acute kidney injury. Demographics collected included sex and age. Comorbidities collected included diabetes and smoking. The American College of Surgeons (ACS) trauma center level (i.e., Level-I vs Level-II) was also collected. Mechanism of injuries included motor vehicle collisions (MVC), bicycle collisions, falls, gunshot wounds, and stab wounds. Data on presentation including hypotension on arrival (defined as systolic blood pressure (SBP) < 90 mmHg), tachypnea on arrival (respiratory rate > 22 breaths/min), tachycardia on arrival (defined as heart rate > 120 beats/min), SBP on arrival, respiratory rate on arrival, and heart rate on arrival were collected. Injury data included GCS score on admission, injury severity score (ISS), and abbreviated injury scale (AIS) > 3 for the head, spine, thorax, and abdomen. Patients who tested positive for alcohol were compared to patients who tested negative using Chi-squared tests for categorical variables and Mann–Whitney *U* tests for continuous variables. Patients aged 13–15 years old were also compared to those 16–17 years old.

Next, univariate analysis for risk of mortality was performed using risk factors determined based on author consensus and a review of the literature, which included severe AIS—head (grade > 3), GCS ≤ 8, presence of midline shift, hypotension, tachycardia, and smoking [4, 19–21]. Next, variables with a moderate correlation with mortality (*p* ≤ 0.2) were included in a multiple logistic regression model to identify independent risk factors for mortality. A blood alcohol concentration (BAC) of greater than 80 mg/dL was compared to concentrations lower than 80 mg/dL in patients with a positive alcohol screen. *p* values were considered statistically significant if < 0.05. The process was repeated for patients aged 13–15 years old and those aged 16–17 years old. This analysis was performed with IBM SPSS Statistics for Windows (Version 24, IBM Corp., Armonk, NY).

## Results

### Demographics, comorbidities, and injury characteristics

A total of 2553 adolescent TBI patients were identified, and 220 (8.6%) tested positive for alcohol. Compared to patients who screened negative for alcohol, patients who screened positive for alcohol were statistically older (15.9 vs. 15.6 years, *p* = 0.001) and had higher rates of smoking (10.0% vs. 5.4%, *p* = 0.005), but were otherwise similar with regards to sex and comorbidities (all *p* > 0.05). ISS and AIS for the head, spine, thorax, and abdomen were similar between the two groups (all *p* > 0.05). Alcohol positive patients had higher rates of stab wounds (1.4% vs. 0.3%, *p* = 0.008) and MVC (58.6% vs. 50.2%, *p* = 0.017), but lower rates of bicycle collisions (6.3% vs. 2.7%, *p* = 0.033). Compared to alcohol negative patients, alcohol positive patients more commonly had a GCS of ≤ 8 on admission (45.4% vs. 38.1%, *p* = 0.036) and had a statistically higher, lowest median heart rate on arrival (98.5 vs. 93.0 beats/min, *p* = 0.016). The two cohorts were otherwise similar with regards to vital signs on arrival (all *p* > 0.05) (Table 1). Compared to patients aged 13–15, patients aged 16–17 had higher rates of diabetes (1.0% vs. 0.2%, *p* = 0.017), hypertension (0.7% vs. 0.1%, *p* = 0.036), and smoking (8.5% vs. 1.8%, *p* < 0.001). In addition, 16–17 year old patients had higher rates of presenting from a MVC (55.8% vs. 43.6%, *p* < 0.001), while 13–15 year old patients had higher rates of presenting as bicycle collisions (9.6% vs. 3.6%, *p* < 0.001), with tachypnea on arrival (20.7% vs. 16.5%, *p* = 0.007), lower median arrival SBP (128 vs. 130, *p* = 0.05), and higher median arrival heart rate (96 vs. 93, *p* = 0.018). Rates of presentation to Level-I trauma centers did not differ between alcohol positive and negative patients nor patients aged 13–15 and 16–17 (Table 2).

**Table 1:** Demographics, comorbidities, and injury characteristics of adolescent traumatic brain injury patients with and without positive alcohol screens

Characteristic	Negative alcohol screen (*n* = 2333)	Positive alcohol screen (*n* = 220)	*p* value
**Age, years, mean ± SD**	**15.6 ± 1.3**	**15.9 ± 1.3**	**0.001**
Male, *n* (%)	1692 (72.5%)	152 (69.1%)	0.277
Comorbidities, *n* (%)			
Diabetes	14 (0.6%)	3 (1.4%)	0.183
**Smoking**	**126 (5.4%)**	**22 (10.0%)**	**0.005**
Mechanism of injury, *n* (%)			
** Motor vehicle collision**	**1172 (50.2%)**	**129 (58.6%)**	**0.017**
** Bicycle collision**	**147 (6.3%)**	**6 (2.7%)**	**0.033**
Fall	312 (13.4%)	30 (13.6%)	0.913
Gunshot wound	194 (8.3%)	15 (6.8%)	0.439
** Stab wound**	**6 (0.3%)**	**3 (1.4%)**	**0.008**
Level-I trauma center, *n* (%)	1218 (52.5%)	101 (45.9%)	0.074
Hypotensive on arrival, *n* (%)	109 (4.7%)	7 (3.2%)	0.310
Tachypneic on arrival, *n* (%)	422 (18.1%)	43 (19.5%)	0.592
Tachycardic on arrival, *n* (%)	383 (16.4%)	37 (16.8%)	0.878
SBP on arrival, median (IQR)	129.0 (25)	128.0 (28)	0.474
Respiratory rate on arrival, median (IQR)	18.0 (5)	18 (6)	0.792
**Heart rate on arrival, median (IQR)**	**93.0 (33)**	**98.5 (32)**	**0.016**
**GCS ≤ 8 on admission,** ***n*** **(%)**	**879 (38.1%)**	**98 (45.4%)**	**0.036**
ISS, median (IQR)	17.0 (17)	17.0 (16)	0.336
AIS (grade > 3), *n* (%)			
Head	1079 (46.2%)	102 (46.4%)	0.974
Spine	26 (1.1%)	3 (1.4%)	0.739
Thorax	124 (5.3%)	8 (3.6%)	0.282
Abdomen	93 (4.0%)	6 (2.7%)	0.355

*A bolded row denotes a variable with p < 0.05, SD* standard deviation, *ISS* injury severity score, *IQR* interquartile range, *SBP* systolic blood pressure, *COPD* chronic obstructive pulmonary disease, *AIS* abbreviated injury scale, *CVA* cerebrovascular accident, *GCS* Glasgow Coma Scale, hypotension is defined as < 90 mmHg, tachypnea is defined as respiratory rate > 22 breaths/min, tachycardia is defined as heart rate > 120 beats/min

**Table 2:** Demographics, comorbidities, and injury characteristics of adolescent traumatic brain injury patients compared by age

Characteristic	Ages 13–15 (*n* = 1022)	Ages 16–17 (*n* = 1531)	*p* value
**Age, years, mean ± SD**	**14.3 ± 0.8**	**16.6 ± 0.5**	** < 0.001**
Male, *n* (%)	739 (72.3%)	1105 (72.2%)	0.941
Comorbidities, *n* (%)			
COPD	8 (0.8%)	7 (0.5%)	0.292
History of CVA	1 (0.1%)	2 (0.1%)	0.813
**Diabetes**	**2 (0.2%)**	**15 (1.0%)**	**0.017**
** Hypertension**	**1 (0.1%)**	**10 (0.7%)**	**0.036**
**Smoking**	**18 (1.8%)**	**130 (8.5%)**	** < 0.001**
Mechanism of injury, *n* (%)			
**Motor vehicle collision**	**446 (43.6%)**	**855 (55.8%)**	** < 0.001**
**Bicycle collision**	**98 (9.6%)**	**55 (3.6%)**	** < 0.001**
Fall	138 (13.5%)	204 (13.3%)	0.897
Gunshot wound	81 (7.9%)	128 (8.4%)	0.695
Stab wound	2 (0.2%)	7 (0.5%)	0.275
Level-I trauma center, *n* (%)	520 (50.9%)	799 (52.2%)	0.517
Hypotensive on arrival, *n* (%)	48 (4.7%)	68 (4.4%)	0.762
**Tachypneic on arrival,** ***n*** **(%)**	**212 (20.7%)**	**253 (16.5%)**	**0.007**
Tachycardic on arrival, *n* (%)	168 (16.4%)	252 (16.5%)	0.989
**SBP on arrival, median (IQR)**	**128 (25)**	**130 (25)**	**0.005**
Respiratory rate on arrival, median (IQR)	18 (6)	18 (5)	0.472
**Heart rate on arrival, median (IQR)**	**96 (31)**	**93 (34)**	**0.018**
GCS ≤ 8 on admission, *n* (%)	404 (40.1%)	573 (37.8%)	0.254
ISS, median (IQR)	17 (16)	17 (19)	0.143
AIS (grade > 3), *n* (%)			
Head	453 (44.3%)	728 (47.6%)	0.109
Spine	7 (0.7%)	22 (1.4%)	0.079
Thorax	49 (4.8%)	83 (5.4%)	0.483
Abdomen	34 (3.3%)	65 (4.2%)	0.239

*A bolded row denotes a variable with p < 0.05, SD* standard deviation, *ISS* injury severity score, *IQR* interquartile range, *SBP* systolic blood pressure, *COPD* chronic obstructive pulmonary disease, *AIS* abbreviated injury scale, *CVA* cerebrovascular accident, *GCS* Glasgow Coma Scale, hypotension is defined as < 90 mmHg, tachypnea is defined as respiratory rate > 22 breaths/min, tachycardia is defined as heart rate > 120 beats/min

### Outcomes

Alcohol positive adolescent trauma patients had a similar mortality rate compared to patients with a negative alcohol screen (13.2% vs. 12.1%, *p* = 0.64). Additionally, there were no differences in hospital LOS (4.0 vs. 4.0 days, *p* = 0.80) and ICU LOS (3.0 vs. 3.0 days, *p* = 0.40) between cohorts. Both groups also had similar ventilator days and complications (all *p* > 0.05) (Table 3). Patients aged 13–15 had a shorter hospital LOS (4.0 vs. 5.0, *p* = 0.002), but similar mortality, ICU LOS, ventilator days, and rates of complications compared to patients aged 16–17 (all *p* > 0.05) (Table 4).

**Table 3:** Clinical outcomes in adolescent traumatic brain injury patients with and without positive alcohol screens

Outcome	Negative alcohol screen (*n* = 2333)	Positive alcohol screen (*n* = 220)	*p* value
LOS, days, median (IQR)	4 (8)	4 (8)	0.800
ICU LOS, days, median (IQR)	3 (5)	3 (6)	0.400
Ventilator days, median (IQR)	3 (6)	2 (6)	0.084
Complications, *n* (%)			
Unplanned ICU admission	23 (1.0%)	4(1.8%)	0.249
Unplanned intubation	26 (1.1%)	1 (0.5%)	0.360
Unplanned return to OR	20 (0.9%)	3 (1.4%)	0.447
Pneumonia/VAP	55 (2.4%)	3 (1.4%)	0.344
Organ space SSI	2 (0.1%)	0 (0.0%)	0.664
Superficial incision SSI	8 (0.3%)	0 (0.0%)	0.384
CAUTI	18 (0.8%)	0 (0.0%)	0.191
CLABSI	7 (0.3%)	1 (0.5%)	0.695
Sepsis	8 (0.3%)	1 (0.5%)	0.789
DVT	18 (0.8%)	1 (0.5%)	0.601
Pulmonary embolism	7 (0.3%)	0 (0.0%)	0.416
Kidney injury	12 (0.5%)	1 (0.5%)	0.905
Mortality, *n* (%)	282 (12.1%)	29 (13.2%)	0.635

*LOS* length of stay, *IQR* interquartile range, *ICU* intensive care unit, *OR* operating room, *SSI* surgical site infection, *VAP* ventilator-associated pneumonia, *CAUTI* catheter-associated urinary tract infection, *CLABSI* central line-associated bloodstream infection, *DVT* deep vein thrombosis

**Table 4:** Clinical outcomes in adolescent traumatic brain injury patients compared by age

Outcome	Ages 13–15 (*n* = 1022)	Ages 16–17 (*n* = 1531)	*p* value
**LOS, days, median (IQR)**	**4 (7)**	**5 (9)**	**0.002**
ICU LOS, days, median (IQR)	3 (5)	3 (6)	0.530
Ventilator days, median (IQR)	3 (5)	3 (6)	0.373
Complications, *n* (%)			
Unplanned ICU admission	8 (0.8%)	19 (1.2%)	0.267
Unplanned intubation	13 (1.3%)	14 (0.9%)	0.387
Unplanned return to OR	7 (0.7%)	16 (1.0%)	0.345
Pneumonia/VAP	18 (1.8%)	40 (2.6%)	0.157
Organ space SSI	0 (0.0%)	2 (0.1%)	0.248
Superficial incision SSI	2 (0.2%)	6 (0.4%)	0.385
CAUTI	6 (0.6%)	12 (0.8%)	0.561
CLABSI	4 (0.4%)	4 (0.3%)	0.564
Sepsis	2 (0.2%)	7 (0.5%)	0.275
DVT	7 (0.7%)	12 (0.8%)	0.776
Pulmonary embolism	2 (0.2%)	5 (0.3%)	0.535
Kidney injury	8 (0.8%)	5 (0.3%)	0.113
Mortality, *n* (%)	115 (11.3%)	196 (12.8%)	0.241

*A bolded row denotes a variable with p < 0.05, LOS* length of stay, *IQR* interquartile range, *ICU* intensive care unit, *OR* operating room, *CVA* cerebrovascular accident, *SSI* surgical site infection, *VAP* ventilator-associated pneumonia, *CAUTI* catheter-associated urinary tract infection, *CLABSI* central line-associated bloodstream infection, *DVT* deep vein thrombosis

### Univariate analysis for risk of mortality

Univariate analysis showed that a severe AIS—head (OR 11.65, CI 8.11–16.73, *p* < 0.001), severe AIS—thorax (OR 3.18, CI 2.14–4.73, *p* < 0.001), severe AIS—abdomen (OR 2.42, CI 1.50–3.89, *p* < 0.001), midline shift (OR 6.06, CI 4.65–7.91, *p* < 0.001), hypotension (OR 12.29, CI 8.31–18.18, *p* < 0.001), tachycardia (OR 3.93, CI 3.03–5.09, *p* < 0.001), and smoking (OR 0.51, CI 0.26–0.97, *p* = 0.041) were associated risk factors for mortality. A positive alcohol screen (OR 1.104, CI 0.73–1.66, *p* = 0.635), severe AIS-spine (OR 2.32, CI 0.98-5.49, *p* = 0.054), and tachypnea (OR 1.11, CI 0.82–1.50, *p* = 0.495) were not associated with mortality risk (Table 5).

**Table 5:** Univariate analysis for risk of mortality in adolescent traumatic brain injury patients with and without positive alcohol screens

Risk factor	OR	CI	*p* value
Positive alcohol screen	1.104	0.733–1.664	0.635
**Severe AIS—head (grade > 3)**	**11.650**	**8.114–16.726**	** < 0.001**
Severe AIS—spine (grade > 3)	2.324	0.984–5.485	0.054
**Severe AIS—thorax (grade > 3)**	**3.181**	**2.138–4.732**	** < 0.001**
**Severe AIS—abdomen (grade > 3)**	**2.416**	**1.501–3.889**	** < 0.001**
**Presence of midline shift on CT scan head**	**6.064**	**4.647–7.913**	** < 0.001**
**Hypotension**	**12.289**	**8.309–18.177**	** < 0.001**
Tachypnea	1.110	0.822–1.498	0.495
**Tachycardia**	**3.928**	**3.031–5.089**	** < 0.001**
**Smoking**	**0.507**	**0.264–0.973**	**0.041**

*A bolded row denotes a variable with p < 0.05, AIS* abbreviated injury scale, *GCS* Glasgow Coma Scale, *CT* computed tomography, hypotension is defined as < 90 mmHg, tachypnea is defined as respiratory rate > 22 breaths/min, tachycardia is defined as heart rate > 120 beats/min

### Multivariable logistic regression analysis for risk of mortality

#### All patients

Multivariate analysis demonstrated that severe AIS—head (OR 4.94, CI 3.26–7.50, *p* < 0.001), GCS ≤ 8 at presentation (OR 33.31, CI 16.81–66.02, *p* < 0.001), midline shift (OR 2.76, CI 2.00–3.83, *p* < 0.001), hypotension (OR 6.99, CI 4.25–11.50, *p* < 0.001), and tachycardia (OR 2.18, CI 1.57–3.30, *p* < 0.001) were associated risk factors for mortality. Smoking was associated with a decreased risk for mortality (OR 0.44, CI 0.20–0.97, *p* = 0.041). A positive alcohol screen was not an independent associated risk factor for mortality (OR 1.24, CI 0.75-2.07, *p* = 0.402 ) (Table 6).

**Table 6:** Multivariable logistic regression analysis for risk of mortality in adolescent traumatic brain injury patients with and without positive alcohol screens

Risk factor	OR	CI	*p* value
Positive alcohol screen	1.243	0.747–2.067	0.402
**Severe AIS—head (grade > 3)**	**4.943**	**3.259–7.498**	** < 0.001**
**GCS ≤ 8**	**33.314**	**16.809–66.024**	** < 0.001**
**Presence of midline shift on CT scan head**	**2.764**	**1.996–3.827**	** < 0.001**
**Hypotension**	**6.994**	**4.253–11.501**	** < 0.001**
**Tachycardia**	**2.182**	**1.569–3.3036**	** < 0.001**
**Smoking**	**0.437**	**0.198–0.966**	**0.041**

*A bolded row denotes a variable with p < 0.05, AIS* abbreviated injury scale, *GCS* Glasgow Coma Scale, *CT* computed tomography, hypotension is defined as < 90 mmHg, tachypnea is defined as respiratory rate > 22 breaths/min, tachycardia is defined as heart rate > 120 beats/min

### Positive alcohol screen

In patients with a positive alcohol screen, BAC > 80 mg/dL was associated with a decreased risk for mortality in a multivariable analysis only including patients with a positive alcohol screen (OR 0.194, CI 0.05–0.84, *p* = 0.029) (Table 7).

**Table 7:** Multivariable logistic regression analysis for risk of mortality in adolescent traumatic brain injury patients with a positive alcohol screen

Risk factor	OR	CI	*p* value
**BAC > 80 mg/dL**	**0.194**	**0.045–0.843**	**0.029**
**Severe AIS—head (grade > 3)**	**10.779**	**1.860–62.453**	**0.008**
**GCS ≤ 8**	**22.165**	**2.577–190.615**	**0.005**
**Presence of midline shift on CT scan head**	**12.290**	**2.939–51.384**	**0.001**
Hypotension	7.556	0.778–73.410	0.081
Tachycardia	3.712	0.785–17.541	0.098
**Smoking**	**0.044**	**0.002–0.949**	**0.046**

*A bolded row denotes a variable with p < 0.05, BAC* blood alcohol concentration, *mg* milligrams, *dL* deciliter, *AIS* abbreviated injury scale, *GCS* Glasgow Coma Scale, *CT* computed tomography

### Ages 13–15 years old

Multivariate analysis demonstrated that in patients 13–15 years old, severe AIS—head (OR 8.46, CI 3.76–19.02, *p* < 0.001), GCS ≤ 8 (OR 83.49, CI 11.41–610.68, *p* < 0.001), midline shift (OR 2.63, CI 1.49–4.62, *p* = 0.001), hypotension (OR 8.91, CI 3.98–19.90, *p* < 0.001), and tachycardia (OR 3.40, CI 1.93–6.01, *p* < 0.001) were associated risk factors for mortality. A positive alcohol screen (OR 1.04, CI 0.42–2.56, *p* = 0.939) and smoking (OR 0.49, CI 0.09–2.65, *p* = 0.41) were not associated with mortality in this age group (Table 8).

**Table 8:** Multivariable logistic regression analysis for risk of mortality in adolescent traumatic brain injury patients aged 13–15

Risk factor	OR	CI	*p* value
Positive alcohol screen	1.036	0.419–2.561	0.939
**Severe AIS—head (grade > 3)**	**8.459**	**3.763–19.017**	** < 0.001**
**GCS ≤ 8**	**83.489**	**11.414–610.683**	** < 0.001**
**Presence of midline shift on CT scan head**	**2.626**	**1.493–4.617**	**0.001**
**Hypotension**	**8.905**	**3.984–19.902**	** < 0.001**
**Tachycardia**	**3.401**	**1.926–6.006**	** < 0.001**
Smoking	0.490	0.091–2.647	0.407

*A bolded row denotes a variable with p < 0.05, AIS* abbreviated injury scale, *GCS* Glasgow Coma Scale, *CT* computed tomography

### Ages 16–17 years old

Multivariate analysis in patients aged 16–17 showed that severe AIS—head (OR 3.84, CI 2.34–6.31, *p* < 0.001), GCS ≤ 8 (OR 27.65, CI 13.22–57.84, *p* < 0.001), midline shift (OR 2.76, CI 1.84–4.15, *p* < 0.001), hypotension (OR 6.20, CI 3.24–11.86, *p* < 0.001), tachycardia (OR 1.80, CI 1.19–2.73, *p* = 0.006), and smoking (OR 0.362, CI 0.15–0.90, *p* = 0.03) were associated risk factors for mortality. A positive alcohol screen (OR 1.35, CI 0.72–2.53, *p* = 0.355) in this age group was not associated with mortality (Table 9).

**Table 9:** Multivariable logistic regression analysis for risk of mortality in adolescent traumatic brain injury patients aged 16–17

Risk factor	OR	CI	*p* value
Positive alcohol screen	1.346	0.717–2.529	0.355
**Severe AIS—head (grade > 3)**	**3.843**	**2.340–6.310**	** < 0.001**
**GCS ≤ 8**	**27.651**	**13.220–57.838**	** < 0.001**
**Presence of midline shift on CT scan head**	**2.764**	**1.843–4.146**	** < 0.001**
**Hypotension**	**6.196**	**3.236–11.862**	** < 0.001**
**Tachycardia**	**1.803**	**1.188–2.734**	**0.006**
**Smoking**	**0.362**	**0.145–0.904**	**0.030**

*A bolded row denotes a variable with p < 0.05, AIS* abbreviated injury scale, *GCS* Glasgow Coma Scale, *CT* computed tomography

## Discussion

In contrast to adult TBI patients, there is limited evidence regarding the effects of alcohol on mortality in adolescent TBI patients. This current national database analysis found no difference in rate and associated risk of mortality for alcohol positive compared to alcohol negative adolescent TBI patients. Furthermore, there was no difference in LOS, ventilator days, or hospital complications between the two groups.

Alcohol use is prevalent among the adolescent trauma population and is a significant concern to public health [22]. Alcohol use during adolescence has been shown to increase risk for TBI [23]. However, the effects of alcohol on mortality in the setting of TBI is unknown. This study found that alcohol was not an independent risk factor for mortality in adolescent TBI patients. A national database study on alcohol and adolescent trauma patients found a higher mortality rate in alcohol positive patients [18]. However, it did not focus on TBI patients and did not control for potential confounders for mortality in TBI including ISS, GCS, and hemodynamic instability. Available literature regarding the effects of alcohol on TBI patients focus on the adult population and have mixed results with regards to mortality [4–15, 23]. One of the most robust studies is a meta-analysis by Raj et al., which evaluated 11 studies and over 95,000 patients and reported that alcohol positive patients had lower mortality compared to alcohol negative patients [24]. A purported mechanism behind the potential neuroprotective effect of alcohol is the inhibition of *N*-methyl-d-aspartate receptor (NMDA)-mediated excitotoxicity in TBI and a reduction of deleterious effects of catecholamine surges [24, 25]. In contrast, higher rates of structural brain atrophy and poorer neurologic outcomes have been found in alcohol positive patients [26]. The developing adolescent brain is markedly different compared to the adult brain and, therefore, may respond differently to alcohol in the setting of injury. Alcohol has been shown to be particularly deleterious in the developing adolescent brain [27]. Experimental studies demonstrated adolescent mice with TBI had higher mortality and worse neurologic outcomes compared to adult mice [28]. The differences in outcomes between our study and those of prior studies in adults may be explained by developmental changes during the adolescent years. Interestingly, we also found that a blood alcohol concentration of greater than 80 mg/dL in alcohol positive patients was protective with regards to mortality. This may indicate that there is a dose-dependent relationship that should be explored further. Further prospective basic science and clinical studies are needed to confirm our findings and continue to uncover the full effects of alcohol across all age groups.

Given the lower rate of mortality overall among adolescent trauma patients compared to adults, studying alcohol’s effects on other outcomes, such as LOS and complications, may be even more important for the adolescent TBI population. This large database study demonstrated that adolescent TBI patients with positive alcohol screens had similar hospital LOS, ICU LOS, ventilator days, and complications compared to patients who screened negative for alcohol. Outcomes specifically among adolescents with TBI for alcohol positive and negative patients have not been previously compared. Aziz et al. studied outcomes in adolescent trauma and found higher rates of acute respiratory distress syndrome (ARDS), pneumonia, DVT, and pulmonary embolism in alcohol positive adolescents [18]. In contrast, our findings are similar to those of Talving et al. and Scheyerer et al. with adult populations who found no difference in hospital LOS, ICU LOS, and complications between alcohol positive and negative TBI patients [5, 11]. It is important to recognize the extensively documented effects of alcohol on long-term outcomes including cognition and social performance [27]. The absence of hospital outcome differences in our study highlights the importance of investigating the long-term effects of alcohol on the developing brain. This study is the first to suggest alcohol may not significantly affect hospital complications and LOS among the adolescent TBI population. This could be useful for predicting outcomes and benchmarking quality of care in the adolescent TBI population.

There are multiple limitations to this study including those inherent to a retrospective database study, which includes misclassification and missing variables. In addition, missing pertinent variables from the TQIP database include blood alcohol concentration, CT imaging findings such as a Rotterdam score, concomitant substance use, history of previous TBI, and hospital alcohol screening policies. Data may not include patients who expired prior to presenting to the hospital. While our multivariable analysis attempted to control for many factors, there still may be potential confounders that are not accounted for and should be addressed in future prospective study. Furthermore, a significant limitation is that the TQIP database is confined to index hospitalization data and lacks crucial post-discharge neurologic outcomes [1, 29]. Specifically cognitive function outcomes such as memory and psychological factors were not available in the database utilized. Finally, as only 220 adolescent patients were alcohol positive, our lack of statistical significance could be the result of limited power.

## Conclusions

This database study demonstrated no difference in rate and associated risk of mortality between alcohol positive and alcohol negative adolescent TBI patients. There was also no difference in LOS or hospital complications between the two groups. The role of alcohol in adolescent TBI is a developing field of research and future basic science studies are needed to elicit the exact impact of alcohol in this population.
